# Music Listening for Supporting Adolescents’ Sense of Agency in Daily Life

**DOI:** 10.3389/fpsyg.2019.02911

**Published:** 2020-01-08

**Authors:** Suvi Helinä Saarikallio, William M. Randall, Margarida Baltazar

**Affiliations:** Department of Music, Art and Culture Studies, University of Jyväskylä, Jyväskylä, Finland

**Keywords:** sense of agency, music listening, everyday life, adolescents, experience sampling

## Abstract

Sense of agency refers to the ability to influence one’s functioning and environment, relating to self-efficacy, and wellbeing. In youth, agency may be challenged by external demands or redefinition of self-image. Music, having heightened relevance for the young, has been argued to provide feelings of self-agency for them. Yet, there is little empirical research on how music impacts adolescents’ daily sense of agency. The current study investigated whether music listening influences adolescents’ perceived agency in everyday life and which contextual determinants would explain such an influence. Participants were 44 adolescents (48% female, 36% with training in music, mean age 14), recruited through local schools. The mobile Experience Sampling app MuPsych was used to collect brief self-reports of personal music listening experiences during daily life. This method assessed the change in the listener’s perceived control over both their emotional states (internal agency), and their external environment (external agency), over 5 min of music listening. Also measured were changes in mood states, and contextual variables (social situation, concurrent activity, and reason for listening). The impact of music on the sense of agency was analyzed using multilevel structural equation modeling. There was no general increase of agency across all music episodes, but agency fluctuations were determined by specific contextual factors. External agency change was predicted negatively by changing environments, while internal agency change was predicted by initial mood and various reasons for listening, including for enjoyment, coping, and enhancing current mood state. Our findings confirmed the plasticity and situational embeddedness of the sense of agency. Music indeed can support agency, but the impact is dependent on a range of situational factors. Sense of agency can be seen as a health resource and significant part of youth development, and current findings provide new insight into when and by which conditions such affordance is likely to be employed.

## Introduction

### Agency: Concept and Definition

Human agency is a core concept in social cognitive theory (Bandura, 1982, 1989, 2006), which posits that people are self-organizing, proactive, self-regulating, and self-reflecting (Bandura, 2006, p. 164). Agency is defined as the ability to influence one’s environment and functioning (Bandura, 1982, 1989). Notwithstanding, individuals are not totally detached from their surroundings; instead, there is a reciprocal causation process, where personal and environmental determinants operate. Agency can, thus, be conceived as “embedded and situated, yet also emergent within historical sociocultural contexts, which in turn are nested in the biological and physical world” (Martin et al., 2003, p.133). Consequently, agency is a plastic, changing, and negotiable stated (as opposed to a trait or competence), reliant on context-bound, interdependent, and dynamic processes (Esser et al., 2016).

Importantly, how successfully people can influence, control, and negotiate events that affect their lives varies from moment to moment, but so do the beliefs in their capability of action (Bandura, 1995). These beliefs are commonly termed as “self-efficacy” (Bandura, 1982), and they influence behavior and its outcomes through several cognitive (e.g., prediction of success/failure), motivational (e.g., resilience), and affective processes (e.g., stress). According to the models of human agency and self-efficacy, every motivator of action and change has in its foundation the core belief that one has the power to effect changes by one’s actions. Simply put, without beliefs of self-efficacy, people do not act on their surroundings or own states. Beliefs of efficacy act as vital forces supporting motivation, well-being, and personal accomplishment in all areas of life (Pajares, 2005, p.339). Indeed, several studies have consistently shown that efficacy beliefs contribute to, for example, smoke-cessation (Schnoll et al., 2011), adherence to healthy behavior (Strecher et al., 1986), recovery from traumatic situations (Bosmans and van der Velden, 2015), and lower levels of depression (Sawatzky et al., 2012).

In sum, agency reveals itself through intentional acts that can produce different outcomes. This can be the case of listening to music: the act of music listening in a given moment and context immediately changes the situation – be it in terms of soundscapes, affective states, connectedness, meanings… (DeNora, 1999, 2000, 2013; Lincoln, 2005; Krueger, 2011, 2018; Skånland, 2013; O’Neill, 2017). In this paper, we conceptualize musical agency as the use of music (in its different genres, behaviors, and contexts…) to capitalize, exercise, and regain agency and sense of agency (similar to “aesthetic agency,” DeNora, 2000, 2001).^1^

### Agency in Adolescence

In many aspects, adolescence is defined by the individuals’ trajectory toward their empowerment as agents who can create and control several aspects of their world (Beyers et al., 2003; Gowers, 2005). As adolescents increase their independence, form their identity, and work on their emotional experiences, they are faced with the opportunity to become self-sufficient agents. During adolescence, agency is practiced throughout almost every dimension of life and a personal growth through mastery is observed. The adolescents’ sense of efficacy is strengthened by learning how to successfully deal with pressing issues (such as vocational definition, sexuality, family, and affect regulation), accompanied by vicarious experiences (such as modeling of a peer’s behavior), persuasive information from others (such as verbal encouragement), and positive physiological feedback (such as states of arousal appraised as confidence) (Bandura, 1977).

Importantly, the sense of agency has been pointed out as a key variable to understand the paths through adolescence into adulthood (Bandura, 1995; Pajares and Urdan, 2006). Thus, it closely relates to several aspects of adolescents’ healthy development and adaptation. Agency is part of the development of behavioral regulation: adolescents’ self-regulation has been for instance defined in terms of three agentic behaviors: selection (choosing opportunities that match with one’s sense of self and personal potential), optimization (refining one’s resources to reach goals) and compensation (being able to “change course” if resources fail) (Lerner et al., 2001). Agency also closely relates to identity formation: a composite measure of agency, consisting of self-esteem, purpose in life, ego strength, and internal locus of control, has been found to positively relate to identity achievement and negatively to identity diffusion (Côté and Schwartz, 2002). Similarly, internal locus of control has been shown to relate to identity achievement while external locus of control relates to identity statuses of moratorium, foreclosure, and diffusion (Lillevoll et al., 2013). It has been argued that the increased pressure in Western societies for each young person to find their own, personal, path to adulthood places agency, self-determination, and self-directedness in a heightened role: those who are able to capitalize on the breadth of opportunities are likely to flourish but those who are unable to exercise personal agency at this stage may find their developmental period particularly distressing (Schwartz et al., 2005). In line with these, the Self-Determination Theory stresses autonomy and competence as essential features for adaptive development and wellbeing (Ryan and Deci, 2000).

### Music, Agency, and Self-Efficacy

A well-established body of literature documents the role of music in youth development (McFerran, 2011; ter Bogt et al., 2012; Miranda, 2013). There is evidence showing that musical behaviors foster the adolescent’s ability to cope with the challenges that matter the most during this developmental stage: identity definition (North and Hargreaves, 1999; North et al., 2000; Hense and McFerran, 2017), connection with peers (Selfhout et al., 2009; Papinczak et al., 2015; ter Bogt et al., 2017), affect regulation (Saarikallio and Erkkilä, 2007; McFerran and Saarikallio, 2014; Leipold and Loepthien, 2015), and shaping of agency (Laiho, 2004; Gold et al., 2011). It has been argued that music occupies such an important place in adolescents’ lives precisely because of the developmental functions it serves (Schwartz and Fouts, 2003; Laiho, 2004; Miranda, 2013). In terms of serving as a personal resource for agency music holds particular potential for the young, as Saarikallio (2019b) points out: “Music is the kids” own world, their playground and kingdom, in which they can shout and be silly, to be fragile and in search of themselves, and make their own, personal, choices.”

As argued by Krueger (2011), music is perceived from birth as a structure that affords possibilities – for constructing and regulating emotions, for expressing and communicating, for shaping relationships and situations. Children’s play, symbols, drawings, and music uses are not only contemplative, but mainly participatory, engaged, and active–i.e., agentic (Karlsen, 2011; Bonsdorff, 2017; Rissanen, 2017; Kuuse, 2018). Through the use of these esthetic resources, children develop from early ages their agency and sense of self-efficacy. In the case of music, it has been observed that there is a constant, informal learning in everyday life (Batt-Rawden and DeNora, 2005), supported by music listening, reflection, and narrative, that create memories, patterns, meanings, and gateways between the individual and the social surroundings. Learning how music can be used empowers the individual to act on their own affective states, wellbeing, health, and agency modes (DeNora, 2000, 2001; Skånland, 2013).

### Music-Fostered Agency and Wellbeing

Seeking and exploring the sense of agency through music seems to be particularly relevant for individuals that are either developing their agency (i.e., youth) or experiencing a reduction in their ability to control their actions and/or environment (e.g., due to illness or challenging personal situations) (Magee, 2017). Batt-Rawden and colleagues have conducted studies with individuals dealing with chronic affective or physical illnesses (Batt-Rawden and DeNora, 2005; Batt-Rawden et al., 2005; Batt-Rawden and Tellnes, 2011). Their data show that, by reflecting on their music uses and learning self-care, the participants increased their sense of control and agency. When using music to cope with pain or stressful medical interventions, control and agency have also appeared to matter significantly: the beneficial effects of music have been reported to be especially visible when the participants could choose their own music to listen to (Mitchell and Macdonald, 2006; Pothoulaki et al., 2008; Bernatzky et al., 2011; Jiang et al., 2013). In a feasibility study of a brief music-based intervention for adolescents, McFerran et al. (2018) observed that the sense of agency was key for the participants: after the intervention, the young participants reported an increased awareness of their position as agents who can utilize the affordances of music to reduce distress and promote their own development.

Sense of agency has been identified as a defining element in music usages and their role on social-emotional health (Saarikallio, 2017, 2019a,b; Saarikallio and Baltazar, 2018). In the conceptual model of health-relevant competencies in music, Saarikallio (2017, 2019a) argues that engaging in musical behaviors can foster emotional health when the emotions expressed, evoked, and regulated through music are surrounded by (a) self-reflective awareness and (b) sense of self-control and agency. Concerning musical self-enhancement, Elvers (2016) proposed a framework according to which there are three main musical experiences bolstering positive self-evaluations: induction of empathy (through identification with the music, self/other merging, and self-confirmation), promotion of social cohesion (through rhythmic entrainment and reinforcement of a cultural worldview and sense of social belonging), and pleasure (through the induction of positive affect, default mode network-activation, and self-referential emotions). According to Elvers, this framework encompasses “the kind of musical experiences that elicit positive affect related to the self, that induce feelings of power and control, that promote positive self-evaluations, and ultimately promote self-esteem” (Elvers, 2016, p. 3). In this line of thought, the use of music to increase the sense of agency and beliefs of efficacy can be seen as one way of musical self-enhancement.

### Aims of the Current Study

Despite the breadth of research arguing that music holds potential for serving as a resource for agency in adolescents’ lives, there is little empirical work on the impacts of music listening to adolescents’ sense of agency. This study was designed to investigate how music listening would relate to the everyday fluctuations of adolescents’ sense of agency.

Following our definition of agency as a plastic and context-bound ability to influence one’s environment and functioning, we placed particular focus on exploring the unfolding situational aspects of daily life as the context in which the impacts of music on agency would occur. We addressed agency both in terms of internal control of one’s feelings and in terms of one’s perceived sense of having control over the external environment (labeled in this paper as internal and external agency). We chose to focus on music listening in contrast to active music making, because that is an activity available practically for all adolescents in the multitude of their everyday life events and environments. Finally, as the majority of current research on music and agency is constituted of theoretical propositions, we decided to provide empirical evidence through measuring changes in agency as a function of daily music listening. Although it has not been observed empirically yet, we expected to find differences in perceived agency after an episode of music listening.

Firstly, we expected to observe a general increase in the self-perceived sense of agency (in terms of both internal and external control) across the episodes of music listening. Secondly, we explored whether the impacts of music listening on the sense of agency would depend on specific contextual factors including simultaneous activities, presence of other people, current mood and energy level, and reasons for music listening at the given moment. Thirdly, we expected changes in both internal and external agency to correlate with changes in valence, with increased sense of agency correlating positively with mood improvement.

## Materials and Methods

### Participants

Participants were recruited through middle schools within the Jyväskylä sub-region of Finland. The research team first contacted the rectors and music teachers of the schools, visited the schools, and provided information about the study to the students as part of their music classes. Students who volunteered downloaded the app with assistance provided by a video instruction and/or the researchers directly. Participants were required to have a mobile phone with the Android mobile operating system, that they used for personal music listening. The sample consisted of 44 year 7 and 8 students (age *M* = 13.67, *SD* = 1.87), all native to Finland, with a mix of both girls and boys (47.7% female). The majority of participants reported no formal practical music training (36.4% reported at least 1 year of training). Participation was incentivized through a draw to win Spotify subscriptions.

### Materials

All research materials were presented through the MuPsych app, which was originally developed and tested by Randall and Rickard (2013), with an updated version developed at the University of Jyväskylä. The app collected data through two means: music experience sample reports (ESRs), and surveys. All items were presented in Finnish. A total of 327 music listening episodes were reported (*M* = 7.43, *SD* = 3.66).

Music experience sample reports were presented immediately when the participant listened to any music on their phone. The initial screen assessed current affective state, with responses given on two 7-point slider scales: the first titled “Mood,” with labels for “Negative,” “Neutral” (center) and “Positive”; and the second titled “Energy,” labeled from “Very low” to “Very high.” These variables were recorded as “initial valence” and “Initial arousal,” respectively. These two dimensions have been demonstrated to be efficient and reliable measures of music induced emotion, explaining a high proportion of variance (Vuoskoski and Eerola, 2011; Thoma et al., 2012). Participants additionally selected one distinct mood that best matched their current feelings and rated how intensely (“Not at all” to “Very”) they felt that mood (named “Mood Intensity”). The following screen consisted of two 7-point slider scales, which were designed to assess the sense of agency: For the external component of agency – a sense of being in charge of the environment – a question was phrased: “*Do you feel that the situation is in your control?*” (from “Not at all” to “Completely”); and for the internal component of agency – focusing on sense of being in charge of one’s feelings – a question was phrased “*Is your mood the way you want it to be?*” (from “Not at all” to “Completely”). The variables were labeled as “External Agency” and “internal agency,” respectively. The following three screens used a list-response format to assess who the listener was with (Listeners), where they were (Location), and what they were doing while listening (Activity).

If music was still playing on the phone after a period of 5 min – as automatically determined by the app – a second series of screens was presented. The first of these screens assessed the same continuous variables measured at the start of listening (Valence, Arousal, Mood Intensity, External Agency, and internal agency), to determine how these changed over the listening episode. The second screen included questions related to the music, on five 7-point slider scales: Mood of the music (subjectively perceived emotion of the music, as distinct from the induced emotion of the listener; Negative – Neutral – Positive), the Energy of the music (Low Energy – High Energy); “*How much attention are you paying to the music?*” (Attention; None – Complete); “*How much are you enjoying this music?*” (Enjoyment; Not at all – Very much); and “*How familiar are you with this music?*” (Familiarity; Not at all – Very familiar). These five music questions were included to provide an overview about how the listener perceived the music at the given episode. The final screens of the music ESR used a branching list-response format to assess the main reason for listening. These reasons were categorized as: For current activity; For entertainment/enjoyment; To relax/calm down; To focus on the Music; For boredom/habit; To cope with a situation; For thinking/reflecting; To raise/boost energy; To enhance my current mood; To diminish my current mood; and To maintain my current mood.

The surveys presented within MuPsych were a series of psychometric scales to assess individual variables, which could be completed at any time. Participants were asked to report their gender and how many years of formal practical music training they had received. They also filled in the 44-item version of the Big Five personality Inventory (John and Srivastava, 1999). Previous research has demonstrated that scales presented within MuPsych produce similar Cronbach alpha scores to those published from the standard questionnaires (Randall and Rickard, 2013).

### Procedure

Prior to the study, an ethics approval was applied for and granted by the University of Jyväskylä Ethical Committee. Information statements were sent to parents of all potential participants through an internal school system, before the participants themselves were contacted during their music classes. They were presented with details about the research and instructed to install the MuPsych app on their mobile phone. All elements of the study were presented through the app, including an information statement, music ESRs, and all surveys. Music ESRs were presented when any music was played on the phone, however, once one had been completed, no more were presented for a period of 4 h, to avoid respondent fatigue. Surveys were presented within the app and could be completed at any time of convenience. This data collection continued for a period of 1 week, after which no more ESRs were presented.

### Data Analyses

Two separate approaches were utilized to analyze the data: aggregate scores on the participant level were used to determine changes in agency overall and across different listening reasons and contexts; and a multilevel model was used to consider context variables, with musical experiences nested within listeners. For the first approach, aggregate scores were created for each individual participant, producing means for valence, arousal, mood intensity, internal, and external agency. This approach is recommended for ESM (Hektner et al., 2007), and has been utilized in previous ESM studies of music use (Randall et al., 2014). Paired-samples *t*-tests were then performed to determine if music listening produced any significant changes in these aggregate scores. The second approach involved the implementation of a multilevel structural equation model (SEM), using Mplus software (version 7.4: Muthén and Muthén, 2015). A total of 327 musical experiences were included in the model, clustered within 44 listeners. This multilevel approach accounted for both the variance in emotional outcomes due to variation amongst musical experiences, and the variance due to variation amongst individual listeners. Maximum likelihood estimation with robust standard errors (MLR) was used, with an accelerated expectation–maximization (EMA) optimization algorithm. Pearson correlation was used to test the relatedness of agency change with valence, arousal, and mood intensity change.

## Results

### Aggregate Analysis

To determine if any overall changes in External Agency, internal agency, Valence, Arousal, or Mood Intensity were produced from music listening, repeated-measures *t*-tests were performed on the aggregate participant scores. These tests revealed no significant changes, with the details reported in Table 1. Also on the aggregate level, no significant effect of gender was found on either external (*p* = 0.239) or INTERNAL AGENCY change (*p* = 0.371), and no significant correlations were observed between these agency measures and BFI personality scales or years of musical training. Aggregate level frequencies revealed that 91% of listening episodes were solitary, 58.11% were at home, 20.42% traveling, and 17.44% at school and the most frequent activity was “Nothing/waiting” 11.72%, followed by 11.15% “In a car,” 11.02% “Focussed listening,” and 10.68% “Studying.” The most frequently reported reasons for listening were “For entertainment/enjoyment” (16.42%), “For current activity” (15.32%), “For boredom/habit” (14.03%), and “To relax/calm down” (12.77%).

**TABLE 1:** Overall changes in agency and affective variables during music listening.

	**Mean**	**Mean**	**Mean**	***t***	***p***
	**(0 min)**	**(5 min)**	**difference**		
External agency	1.879	1.975	0.096	1.184	0.246
Internal agency	0.985	1.135	0.150	1.628	0.115
Valence	1.238	1.334	0.096	0.933	0.359
Arousal	0.213	0.429	0.217	1.901	0.068
Mood intensity	1.697	1.418	−0.280	−2.457	0.020^∗^

*^∗^is significant at point 0.05.*

### Multilevel Analysis

Correlations between changes in External Agency, internal agency, Valence, Arousal, and Mood Intensity are displayed in Table 2. This shows significant positive correlations between all variables, with the strongest between internal agency change and Valence change. In line with the hypothesis, valence change and agency change were positively correlated.

**TABLE 2:** Correlations between agency change variables and affective change variables.

	**Internal**	**Valence**	**Arousal**	**Mood**
	**agency**	**change**	**change**	**intensity**
	**change**			**change**
External agency change	0.197^∗∗^	0.186^∗∗^	0.147^∗∗^	0.017
Internal agency change	–	0.293^∗∗^	0.142^∗∗^	−0.105^∗^

*^∗^is significant at point 0.05 and ^∗∗^is significant at point 0.01.*

A multilevel structural equation model was constructed to assess the interaction of all measured variables, with all significant predictors presented in Table 3 (with the effects of initial agency states on their respective changes in agency removed). This model produced a standardized root mean square residual (SRMR) of 0.038 on the musical experience level (with values of less than 0.08 generally considered a good fit: Hu and Bentler, 1999).

**TABLE 3:** Structural equation model predictors of agency change.

**Outcome**	**Predictor**	**β**	***SE***	**β*/SE***	***p***
External agency change	Activity: Walking	−0.112	0.051	−2.20	0.028
	Activity: On transport	−0.080	0.040	−2.04	0.042
Internal agency change	Initial valence	0.306	0.084	3.62	<0.001
	Reason: Enjoyment	0.200	0.085	2.37	0.018
	Reason: To cope	0.153	0.068	2.26	0.024
	Reason: Enhance mood	0.152	0.053	2.87	0.004
	Listening alone	0.128	0.062	2.06	0.040
	Activity: Studying	−0.113	0.046	−2.49	0.013
	Reason: Improve mood	0.085	0.041	2.07	0.039
Music valence	Initial valence	0.310	0.105	2.95	0.003
	Location: Home	0.160	0.072	2.23	0.026
	Activity: Walking	0.124	0.058	2.15	0.031
	Activity: Housework	0.114	0.040	2.85	0.004
	Activity: Studying	0.108	0.049	2.22	0.026
Music arousal	Initial valence	0.322	0.105	3.05	0.002
	Activity: Focussed listening	0.144	0.070	2.07	0.039
	Activity: Housework	0.124	0.045	2.76	0.006
Attention to music	Listening alone	0.191	0.083	2.32	0.020
	Initial internal agency	0.186	0.089	2.09	0.037
	Reason: Improve mood	0.144	0.061	2.37	0.018
	Activity: Housework	0.120	0.049	2.46	0.014
Familiarity of music	Listening alone	0.193	0.088	2.19	0.028
Reason: Enjoyment	Initial arousal	−0.213	0.076	−2.80	0.005
	Activity: Going to sleep	−0.146	0.043	−3.42	0.001
Reason: Diminish mood	Initial internal agency	−0.210	0.095	−2.21	0.027
Reason: Enhance mood	Initial valence	−0.312	0.089	−3.49	<0.001
	Initial external agency	0.228	0.072	3.18	0.001
	Initial internal agency	0.171	0.055	3.10	0.002
Reason: Focus on the music	Activity: In a car	−0.194	0.087	−2.24	0.025
	Initial external agency	−0.169	0.085	−1.99	0.047
	Listening alone	0.163	0.065	2.49	0.013
Reason: For activity	Activity: Focussed listening	−0.168	0.054	−3.10	0.002
	Activity: Eating	−0.126	0.032	−3.92	<0.001
	Activity: Doing nothing	−0.116	0.058	−1.99	0.046
Reason: Improve mood	Location: Home	0.216	0.082	2.62	0.009
Reason: Maintain mood	Activity: Eating	−0.055	0.024	−2.33	0.020
Reason: Raise energy	Location: Traveling	−0.334	0.100	−3.35	0.001
	Location: Home	−0.250	0.111	−2.25	0.025
	Listening alone	−0.193	0.054	−3.56	<0.001
	Location: Work/school	−0.166	0.070	−2.38	0.018
	Activity: On transport	0.153	0.049	3.15	0.002
	Activity: In a car	0.110	0.049	2.25	0.024
Reason: Thinking	Activity: Gaming	−0.061	0.025	−2.47	0.014
Reason: To cope	Activity: Walking	0.166	0.082	2.03	0.043
	Location: Work/school	0.145	0.057	2.53	0.011
	Activity: Studying	−0.113	0.055	−2.06	0.039
	Activity: Going to sleep	−0.104	0.049	−2.13	0.034
	Activity: Eating	−0.088	0.024	−3.73	<0.001
Reason: To relax	Listening alone	−0.252	0.071	−3.53	<0.001
	Activity: Going to sleep	0.184	0.081	2.28	0.023
	Activity: Focussed listening	0.134	0.049	2.72	0.006
	Activity: On transport	−0.128	0.038	−3.32	0.001

*β, standardized parameter estimate; *SE*, standard error; β/*SE, t* value associated with the parameter estimate; *p*, two-tailed *p* value (only <0.05 shown).*

These results in Table 3 reveal two weak negative predictors for External Agency change: the activities of walking (β = −0.112; frequency of walking experiences = 5.11%), and on transport (“On bus/train/plane”; β = −0.080; frequency 2.18%). internal agency change was positively predicted by initial valence of the listener (β = 0.306), the reason “Enjoyment” (“For entertainment/enjoyment”; β = 0.200; frequency 16.42%), the reason “To cope” (“To cope with a situation”; β = 0.153; frequency 4.35%), the reason “Enhance mood” [“To feel more (of my selected mood state)”; β = 0.152; frequency 2.75%], listening alone (β = 0.128; frequency 91.00%), and negatively predicted by the activity of studying (β = −0.113; frequency 10.68%).

## Discussion

The analysis of the music listening experiences as a whole revealed that changes in the sense of agency are not transversal to all episodes. However, fluctuations in agency were observed within the episodes and particular context factors and reasons for listening were identified as explaining the modulations in perceived agency. These results are in line with both music literature and agency literature. The former informs us that there is a plethora of contextual factors that shape the usages and related affective impact of music in everyday life listening (Randall and Rickard, 2017a, b), and the latter posits that the sense of agency is embedded, context-bound, plastic, and malleable (Martin et al., 2003; Esser et al., 2016). The findings of the current study confirm that the relationship of music and perceived agency is intrinsically embedded in the multifaceted nature of our daily experiences, dependent on specific contextual variables. Additionally, the current results confirm the feasibility of measuring fluctuations in perceived agency, even after a short period of music listening (5 min).

The two components of agency – internal and external – were predicted by a distinctively different set of contextual determinants. Significant predictors of external agency were relatively straightforward, with walking and transportation negatively predicting increases in external agency. This is illustrative of external agency being dependent on objective, environmentally defined, constraints, and activities rather than internal experiences. These effects may be less dependent on the music listening experience itself, as we would expect control over our external world to decrease when traveling through different environments, regardless of music-related variables. Previous studies have looked into the motivations and outcomes of listening to music in situations where there is little or no control over our surroundings, such as traveling (Heye and Lamont, 2010; Skånland, 2013), driving (Dibben and Williamson, 2007), and working in an office environment (Haake, 2011). It has been found that listeners envelop themselves in their personal music, with high levels of attention to the music itself, but also increased awareness of their external environment (cf. “auditory bubble,” Bull, 2006; Heye and Lamont, 2010). Listening to music when the external sense of agency is low can be seen as an attempt at managing inner/outer spaces, avoiding stressors from the environment, and self-regulating. Indeed, our results confirm that when initial external agency was low, listeners were more likely to be listening for the reason “to focus on the music.” This suggests that if listeners have little control over their external environment, the music itself becomes important to them; they feel the need for the “auditory bubble.” The reason for listening “to cope” is of particular relevance here, as it was utilized more frequently by those who were walking, and in turn positively predicted internal agency. Worded as “to cope with a situation,” this suggests that listeners were using music to improve their internal mood state to deal with their lack of control over the external situation. Furthermore, previous research using mobile ESM found this specific reason to be related to low valence, low arousal moods, as well as poor emotional health and well-being of the listener (Randall and Rickard, 2017b). These external agency findings also resonate with a concept that Krueger calls the musically extended mind (2014). Music can be seen as an external resource that enhances internal abilities, and affords possibilities for action at the affective, physical, and social levels (Krueger, 2011, 2014). In a way, this kind of focused music listening has the potential to alter the external environment and the individual’s position in it, thus creating a sense of agency (DeNora, 1999, 2000; Krueger, 2014).

In contrast, change of internal agency was significantly predicted by several mood-related experiences, reasons, and goals – in a word, internal experiences. High initial levels of internal agency also predicted greater attention to music, a direct contrast to external agency: engaged (focused, attentive) listening was predicted by *low* control of the situation but by *high* contentment of the internal experience. In general, the fact that internal agency change was positively predicted by specific internal reasons for listening suggests that people intend to use music in certain ways that will change their mood to the way they want it to be. This concept is strongly supported by the music psychology literature (Thayer et al., 1994; DeNora, 1999; Saarikallio, 2011; Van Goethem and Sloboda, 2011; Baltazar and Saarikallio, 2016; Baltazar, 2019), including previous mobile ESM research that has found that emotional reasons for listening on mobile phones are utilized to fulfill specific emotional needs (Randall and Rickard, 2017b). The model results show that when internal agency is low (mood state is not ideal), people more frequently intend to diminish the intensity of their current mood state while when internal agency is high (mood state is ideal), people more frequently intend to enhance the intensity of their current mood state, which in turn predicts an increase of internal agency. Overall, it seems that people deliberately use music to control their internal states and this successfully results in an increased sense of internal agency.

Internal agency change was particularly predicted by positive initial valence and reasons for listening that related to positive mood maintenance or mood repair: entertainment/enjoyment, mood enhancement, mood improvement, and coping. Prior research has identified entertainment and positive mood maintenance as one of the most common motivations for music listening for adolescents and young adults (North et al., 2000; Saarikallio, 2008; Randall and Rickard, 2017b). Entertainment has previously been linked to raising moods while listening to music alone (Saarikallio and Erkkilä, 2007) and to higher wellbeing and lower symptomatology (Gebhardt and von Georgi, 2007; Thomson et al., 2014), and the use of music as a coping resource in general has widely been acknowledged in the recent literature (e.g., Miranda and Claes, 2009; Skånland, 2011; Van Goethem and Sloboda, 2011; Miranda, 2019). Entertainment has been considered a regulation strategy of affect that is more embodied and pleasure-oriented, while coping has been seen more closely linked with mental contemplation and the use of cognitive resources: these two types of listening have been argued to serve as complementary components of affect self-regulation (Baltazar and Saarikallio, 2019). Our results show both aspects being predictive of internal agency change, demonstrating that internal agency can be achieved through both types of affect-regulatory acts.

A noteworthy observation is that while mood enhancement was predicted by both high initial internal and external agency, the strongest predictor of mood enhancement actually was low initial valence. This indicates that adolescents sometimes want to enhance their current negative mood. This deliberate maintenance of a negative state through music listening has been identified in previous research and discussed in relation to the concept of “misery-sharing” (Gibson et al., 2000; Zillmann and Vorderer, 2000; Skånland, 2013). Whether the purposeful enhancement of negative mood increases internal agency remains a bit ambiguous: Mood enhancement did predict internal agency. However, the expected positive correlation between valence increase and internal agency increase would indicate that the increase of internal agency relates to mood improvement, not to the enhancement of negative mood. Another noteworthy observation was that internal agency change correlated negatively with mood intensity change. The correlation was relatively low but suggests that decrease of mood intensity may relate to increase of internal agency. Previous research has demonstrated emotional stability as a determinant of affective wellbeing (John and Srivastava, 1999; Tomyn and Cummins, 2011), while adolescence has been characterized by higher emotional intensity and lower stability (e.g., Larson et al., 2002). Due to the low effect size this finding should be interpreted with some caution, but future research on how internal agency may correlate with attempts toward emotional homeostasis might be fruitful for understanding music as agentic mood management.

Overall, our results on internal agency and mood changes might be partly explained by the general reciprocal connection between positive experiences, self-efficacy, and sense of agency. As mentioned previously, self-efficacy is the belief that one is able to achieve a certain goal. This belief is reinforced by positive experiences and, in turn, promotes a sense of agency and action-taking. Thus, a positive initial valence might have fostered the participants’ sense of agency on their own feelings. This process is better captured by the correlations observed between agency (internal and external) and changes in mood (valence, especially, but arousal also). Feeling better or feeling more of a certain positive affective state might have been experienced as a successful event by the participants. Generally, experiences of mastery and accomplishment serve as a boost to the adolescents’ perception of self-efficacy, which in turn inform the perception of agency in the short-term and foster the development of agency in the medium- and long-term (Bandura, 1982, 2005; Zimmermann and Cleary, 2005). Music has been proposed as one resource that successfully leads to feeling empowerment (i.e., the reinforcement of efficacy beliefs; Wallace-DiGarbo and Hill, 2006) and self-enhancement (i.e., the tendency to evaluate oneself positively; Elvers, 2016). Saarikallio et al. (2018) found that power-related emotions (self-determination, independence, daring, freedom, strength, empowerment, rebelliousness, and unconstrained feeling) were the second strongest factor out of six explaining music-related *pleasure* in the participants’ everyday life. According to the authors, the factor *power* reflects health-fostering experiences and is closely related to the sense of agency given the self-determination and control it implies. The observed relatedness of agency increase with valence increase is a noteworthy addition to the broader literature on music and wellbeing, proposing that agency and mood regulation may serve as dialogical determinants for adaptive and health-fostering music engagement (Saarikallio, 2019a).

Internal agency also increased when the adolescents were listening to music *alone*. This further solidifies the linkage of internal agency to personal and engaged listening. Furthermore, the act of solitary listening can be seen as an important part of youth development in particular. During youth, solitude becomes a constructive part of daily life, supporting the development toward independence, with solitary music listening emerging as an opportunity for self-exploration and personal mood regulation (Larson, 1995). When listening to music on their own, adolescents often report that they create their own world where they can work their affective material (e.g., Saarikallio and Erkkilä, 2007; Van Goethem and Sloboda, 2011). Music also seems to afford precious possibilities for moving between separation and connectedness (DeNora, 2000; Karlsen, 2011). One might argue that this context (listening alone) fosters their autonomy and agency as important steps and outcomes of their development. In developmental psychology, it has been suggested that agency is the marking factor in healthy paths through adolescent, rather than independence, separation, and autonomy from parents (Beyers et al., 2003). Yet, according to Beyers and colleagues, separation might be a needed step at the onset of adolescence, with the goal of developing as a self-direct and, importantly, connected agent.

In line with the social cognitive theory and agency literature, the two aspects of agency measured in the current study – internal and external sense of agency – were correlated, but with relatively low effect size. Both components are closely interconnected in the experience of agency, but, notwithstanding, may fluctuate somehow independently based on the context and on the relevance of internal or external factors in the particular situation (Bandura, 1982, 1989). At this point, it is not possible to discern whether the correlation between the internal and external sense of agency is situated at the goal or at the outcomes level (i.e., were the adolescents seeking to regulate their agency globally or did the change in one aspect of agency foster the change in the other aspect). Further investigation would be needed to explore the internal structure of self-perceived agency in adolescents’ daily use of music. Nonetheless, it is noteworthy that the two aspects of agency were related to music listening behaviors in a differentiated manner in terms of the contextual determinants. Even though agency in the context of music education and art programs is commonly addressed as a homogeneous concept, at least in the case of music listening it might be useful to explore separately the sense of agency related to inner mood states and the sense of agency related to the surrounding context.

There are certain limitations to consider when interpreting the current results. Firstly, the study involved the subjective assessment of various internal and emotional states, which may be vulnerable to a range of confounding factors. These issues may be amplified for young adolescents, who may have individual sensitivity issues or difficulty in identifying and reporting their emotional states. This subjective self-reporting may also be susceptible to the somewhat intrusive nature of event-based sampling, which could influence the reported emotions. The concepts of internal and external agency were measured only with single items, thus reflecting a relatively narrow definition of each. While this was necessary in the current study in order to keep the episodes answerable when the same questions were answered several times a day, it would be important to encourage future research to take a more elaborate qualitative approach on the conceptual definition of music-based agency and its potential subcomponents. Furthermore, the timeframe over which changes in these variables were measured was limited to 5 min. The results suggest that this was adequate time to elicit changes in agency, but a longer measurement period in future research would yield further insight into how agency fluctuates over time. Finally, the relatively small sample size of the current study was appropriate for exploring contextual variation between the episodes but did not allow modeling the larger interplay of the musical, individual, and contextual determinants. Collecting larger samples for the inclusion of individual differences or using objective measures for assessing musical content might be interesting avenues for future research.

## Conclusion

In conclusion, it appears that music listening can indeed support adolescents’ sense of agency in daily life, but this clearly is not a phenomenon that can be averaged across all situations and for all listeners, or even for all subcomponents of agency. Our ESM approach was able to identify particular contextual predictors for the impact of music listening on internal and external agency within the plethora of everyday experiences. As would be expected, External Agency was decreased when listeners were traveling through different environments, either on foot or in transport. In contrast, internal agency was increased when the initial mood of the listener was positive, and also when specific reasons for listening were used, suggesting that this outcome is more dependent on the deliberate use of music to influence mood. Overall, the findings provide pioneering insight into understanding music listening from the perspective of adolescent agency and wellbeing within the complexity of daily experiences.

## Data Availability Statement

The datasets generated for this study are available on request to the corresponding author.

## Ethics Statement

The studies involving human participants were reviewed and approved by the University of Jyväskylä Ethics Board. Informed consent from the participants’ legal guardian/next of kin was obtained by pressing the accept button in the data collection interface of the mobile phone.

## Author Contributions

SS, WR, and MB conceived the study, developed the design, conducted the participant recruitment and data collection, compiled the literature, and wrote the manuscript. SS and MB drafted the items. WR set up the data collection app and conducted the data analyses. SS and WR acquired the ethical clearance. All authors reviewed and approved the final version of the manuscript.

## Conflict of Interest

The authors declare that the research was conducted in the absence of any commercial or financial relationships that could be construed as a potential conflict of interest.

## Footnotes

1 Different from “musical agency” used in the context of music education by Wiggins (2016) as a “sense that they can initiate and carry out their own musical ideas and ideas about music.”
